# Effects of caffeinated chewing gum on prolonged repeated-sprint performance in normobaric hypoxia

**DOI:** 10.3389/fnut.2026.1954306

**Published:** 2026-09-16

**Authors:** Jin Huang, Yuean Yang, Ruiguo Xue, Linyin Xu, Jingxin Liu, Jue Liu, Yinhang Cao

**Affiliations:** 1 School of Athletic Performance, Shanghai University of Sport, Shanghai, China; 2 Department of Rehabilitation Medicine, Huashan Hospital, Fudan University, Shanghai, China; 3 Faculty for Physical Education, Shanghai International Studies University, Shanghai, China; 4 School of Physical Education, Shanghai University of Sport, Shanghai, China

**Keywords:** caffeinated chewing gum, ergogenic aids, hypoxia, repeated-sprint ability, team sports

## Abstract

**Purpose:**

This study aimed to investigate the effects of caffeinated chewing gum on performance during a prolonged intermittent-sprint test (IST) under normobaric hypoxia.

**Methods:**

Ten team-sport athletes (six men and four women) participated in a double-blind, randomized crossover trial with a counterbalanced treatment order, chewing either caffeinated gum (6 mg/kg) or placebo gum on two separate occasions, 7 days washout period. After chewing for 10 min, participants performed an IST protocol in hypoxia (inspired oxygen fraction: 16.5 ± 0.2%, ~2,000 m). The IST consisted of two 40-min halves separated by a 15-min halftime recovery, each comprising twenty 2-min blocks with an 8-s all-out sprint, 100 s of active recovery at 35% of peak oxygen uptake, and 12 s of passive rest. Total work done, power output, muscle and cerebral oxygenation were recorded.

**Results:**

Total work done was significantly greater with caffeinated gum, increasing by 4.4% in the first half [mean difference = 6 kJ, 95% CI (2.71, 10.08)] and a 2.9% increase in the second half [mean difference = 4 kJ, 95% CI (1.18, 6.57)] compared to placebo (all *p* < 0.05). Caffeinated gum also significantly increased both peak and mean power output (all *p* < 0.05). Cerebral total hemoglobin and oxyhemoglobin were higher in both halves with caffeinated gum, while muscle deoxyhemoglobin and reoxygenation capacity were significantly higher in the first half compared to placebo (all *p* < 0.05).

**Conclusion:**

Caffeinated chewing gum (6 mg/kg) enhances prolonged repeated-sprint performance under normobaric hypoxia, accompanied by increased cerebral oxygenation and muscle reoxygenation capacity.

## Introduction

Most team sports (e.g., football, rugby, and field hockey) require athletes to repeatedly perform high-intensity efforts interspersed with brief recovery periods throughout competition (1–3). The ability to recover and maintain sprint performance during successive efforts is therefore considered a key determinant of performance in intermittent sports (4, 5). However, when athletes compete or train under hypoxic conditions, reduced oxygen availability places additional physiological stress on repeated-sprint exercise (6–8). Previous studies have demonstrated that hypoxia accelerates performance decline during repeated sprints, which may be associated with impaired cerebral oxygenation and reduced muscle reoxygenation during recovery periods (6, 9, 10). Therefore, identifying effective nutritional strategies capable of preserving repeated-sprint performance under hypoxic conditions has important practical implications for team-sport athletes.

Caffeine is one of the most widely consumed nutritional ergogenic aids, with established benefits for endurance (11, 12), team sports (13), and resistance exercise performance (14, 15). Our previous study demonstrated that caffeine ingestion (6 mg/kg) improved total work and power output during a prolonged intermittent-sprint test (IST) under normobaric hypoxia (2 × 40-min halves simulating a team-sport match) (16). However, it should be noted that this study employed a traditional capsule form of caffeine, which typically requires ingestion 45–60 min prior to exercise to allow sufficient absorption and metabolism (17, 18), which may limit its practicality for rapid pre-competition supplementation or for substitute players entering play in team sports. As a novel caffeine delivery strategy, caffeinated chewing gum offers an alternative supplementation approach by enabling rapid caffeine absorption through the oral mucosa, thereby facilitating faster caffeine availability during exercise (18). Furthermore, previous studies have reported beneficial effects of caffeine chewing gum on various types of exercise performance [e.g., endurance (19), sprint (20), and muscular power performance (21)] under normoxic conditions. Nevertheless, whether this rapidly absorbed caffeine formulation can effectively preserve prolonged IST performance under hypoxic conditions remains unknown. Furthermore, as acknowledged in the previous study (16), the lack of near-infrared spectroscopy (NIRS) measurements limited our ability to determine whether the caffeine-induced improvements in power output during prolonged IST were associated with enhanced cerebral oxygenation and muscle reoxygenation responses.

Therefore, the aim of the present study was to investigate the effects of caffeinated chewing gum (6 mg/kg) on performance during a prolonged IST under moderate normobaric hypoxia with NIRS measurements. We hypothesized that caffeinated chewing gum would enhance prolonged repeated-sprint performance under normobaric hypoxia, with enhancements in cerebral oxygenation and muscle reoxygenation capacity. This study provides insights into potential oxygenation-related responses associated with caffeine supplementation and may contribute to optimizing caffeine delivery strategies for athletes performing in hypoxic environments.

## Materials and methods

### Ethical approval

This study was approved by the Shanghai University of Sport Ethics Committee (No. 102772025RT099) and conducted in accordance with the latest revision of the Declaration of Helsinki, with each participant providing verbal and written informed consent.

### Participants

An *a priori* power analysis conducted using G^*^Power software (version 3.1.9.7; Heinrich-Heine-Universität Düsseldorf, Düsseldorf, Germany) indicated that a minimum of 8 participants was required (alpha: 0.05, power: 0.80, correlation coefficient: 0.5, and effect size = 0.5 for total work done as reported by Schneiker et al. (22), who observed an 8.5% improvement in total sprint work with 6 mg/kg caffeine). Considering potential dropouts, ten college team-sport athletes (six men and four women) from basketball, volleyball, and soccer (college-level competitive status; ≥3 years of formal competitive training; weekly training volume: 6–10 h, including scheduled competitive matches) were recruited during the competitive season. Participant characteristics were as follows: age, 22.2 ± 2.1 yr; height, 178.7 ± 6.0 cm; body mass, 75.0 ± 7.1 kg; peak oxygen uptake (V°O_2peak_), 50.0 ± 2.5 ml·kg^−1^·min^−1^. These participants were recruited specifically for the present study and did not overlap with those included in our previous investigation (16). All female participants reported regular menstrual cycles (24–32 days) and were not using hormonal contraceptives. For each female participant, both experimental trials were scheduled during the early follicular phase (days 2–7 of the menstrual cycle) to minimize potential hormonal influences on caffeine metabolism and exercise performance. All participants were non-smokers, free from prescription medication use, and had not undertaken altitude training within the preceding 6 months. Habitual caffeine intake (45.5 ± 40.1 mg/day) was assessed using a validated self-reported questionnaire (23), and all participants were categorized as either caffeine-naive or mild caffeine consumers (0–2.99 mg/kg/day) (24). None of the participants withdrew during the entire experiment.

### Experimental overview

Each participant visited the laboratory on three separate occasions, which included one pre-experimental and two experimental sessions. During the pre-experimental session (visit 1), participants performed an incremental cycling test, followed by familiarization to the main experimental protocol (see “*Incremental cycling test and familiarization session*” section). At least 2 days later, participants completed two experimental trials (visits 2 and 3) in a randomized, double-blind, placebo-controlled, counterbalanced crossover design, with a 7-day washout period between trials (22, 25). The treatment sequence was generated by an independent researcher using a web-based random number generator (www.randomizer.org) and was counterbalanced across participants, with five participants assigned to each sequence (Sequence A: placebo → caffeine; Sequence B: caffeine → placebo). The same independent researcher, who was not involved in data collection or analysis, prepared opaque, identically matched gum pellets (caffeine or placebo), assigned participants to the treatment sequences, and maintained allocation concealment by labeling each trial with a unique alphanumeric code. Participants, investigators, and the researcher responsible for data analysis remained blinded to treatment allocation until the final data lock. Linear mixed-effects models were used to assess potential period, sequence, and carryover effects, with treatment, period, and sequence specified as fixed effects and participant specified as a random effect. No significant period, sequence, or carryover effects were detected, supporting the validity of the crossover design. All trials were conducted at the same time of day under controlled laboratory conditions (21 ± 1.5 °C and 57 ± 3% relative humidity). On testing days, participants were instructed to drink 500 ml of water 2 h before each test to ensure euhydration. On arrival at the laboratory, they voided their bladders and body mass was measured. After instrumentation, participants chewed caffeine-containing gum or a matched placebo gum for 10 min at 15 min before exercise (see “*Supplementation protocol*” section). Following the 10 min gum-chewing period, the participants entered a normobaric hypoxic tent (At-Home Cubicle Altitude Tent Standard, Hypoxico, USA) to prepare for the IST (see “*Intermittent sprint protocol*” section below).

### Dietary and exercise control

Participants were asked to keep a food and activity diary for the 48-h period before each test and were asked to avoid alcohol and vigorous exercise during this time. Participants were given a list of caffeine-containing foods and beverages and were instructed to abstain from consuming these products from 48 h preceding the testing session and throughout the experimental trials. To verify compliance with caffeine abstinence, participants were instructed to record any caffeine consumption in their diaries, and any reported caffeine intake would have resulted in exclusion from the study (none were excluded). On testing days, participants arrived at the laboratory following an overnight fast of at least 8 h, having consumed their last meal at least 3 h before arrival. The timing and composition of the final meal before each trial were recorded and replicated before the second trial. Additionally, the 48-h food and activity diaries were used to ensure consistency in dietary intake and physical activity patterns between the two experimental trials. On testing days, participants consumed no food or beverages before the test (other than what was provided by the experimenters) following arrival at the laboratory.

### Incremental cycling test and familiarization session

V°O_2peak_ was determined using an incremental cycling protocol previously employed by our group (26). Expired gases were measured breath-by-breath using a metabolic gas analysis system (Metalyzer^®^3B; Cortex, Leipzig, Germany). V°O_2peak_ was defined as the highest 30-s average value achieved before volitional exhaustion. Power output and V°O_2_ data from this test were used to determine the exercise intensity for the IST (27). Following a 20-min recovery period, participants performed a 20-min familiarization IST consisting of ten 2-min blocks, each comprising an 8-s sprint, 100-s active recovery at 35% V°O_2peak_, and 12-s passive rest. In addition, seat and handlebar positions were adjusted according to preference, and replicated during all experimental trials. The same electronically braked cycling ergometer (Lode Excaliber Sport, Groningen, Netherlands) was used throughout the experiment, and participants' attire (i.e., shorts, socks and sport shoes) was kept consistent as well.

### Altitude simulation

The normobaric hypoxic tent (183 × 245 × 245 cm) was used to simulate a hypoxic environment equivalent to ~ 2,000 m above sea level (inspired O_2_ fraction: 16.5 ± 0.2%), achieved through a hypoxic gas generator (HYP123 Altitude Generator, Hypoxico, USA) (25). Given that matches of the 2026 FIFA World Cup will be played in Mexico City (~2,240 m), players may be exposed to moderate-altitude hypoxic environments during competition. Therefore, the present study implemented moderate-altitude conditions corresponding to terrestrial elevations of approximately 1,800–2,200 m (28–30).

### Supplementation protocol

Ten team-sport athletes (6 men and 4 women) participated in a double-blind, randomized crossover trial, chewing either caffeinated gum (6 mg/kg caffeine) or placebo gum during two experimental sessions separated by a 7-day washout period. The caffeine dose of 6 mg/kg was selected based on previous studies demonstrating the ergogenic effects of this dose on repeated-sprint performance under normobaric hypoxia (16, 25). Caffeinated and placebo gums were commercially available products: Military Energy Gum (Market Right, Inc., Plano, IL, USA) and non-caffeinated chewing gum (Spearmint Extra Professional; Wrigley's, Chicago, IL, USA), respectively. Each piece of Military Energy Gum contained 100 mg of caffeine according to the manufacturer's specifications. The target caffeine dose (6 mg/kg) was individually calculated based on each participant's body mass, and the corresponding amount of caffeinated gum was prepared for administration. As the manufacturer specifies caffeine content per whole gum piece rather than per fraction, caffeinated gum was thoroughly ground and kneaded before portioning to minimize potential variability in caffeine distribution within the gum matrix. The prepared gums were formed into small boluses with consistent appearance and size before administration, and placebo gum underwent the identical preparation procedure. Both gums had a similar spearmint flavor and were prepared using the same procedures to minimize potential differences in sensory characteristics and handling between conditions. The prepared gum portions were weighed using a precision digital scale (accuracy: ±0.001 g) and placed into identical opaque containers labeled with anonymous treatment codes by an investigator who was not involved in participant testing or data collection. Participants and investigators responsible for outcome assessment remained blinded to treatment allocation throughout the experimental procedures. Randomization codes were not disclosed until completion of all experimental procedures and data analyses. Following the final experimental trial, participants were asked to identify their perceived treatment allocation to assess the success of blinding. Only 4 of 10 participants correctly identified the treatment order (binomial test, *p* = 0.38), indicating that blinding was successfully maintained. No adverse responses to either caffeine gum or placebo were reported.

### Intermittent sprint protocol

Participants first performed a standardized warm-up consisting of 3 min of cycling at 50% V°O_2peak_, followed by two 1-min periods alternating 30 s at 70% V°O_2peak_ with 30 s of rest. After a 2-min seated recovery, the IST commenced. The IST comprised two 40-min halves separated by a 15-min halftime recovery (31). Each half consisted of twenty consecutive 2-min blocks, with every block including an 8-s maximal sprint, 100 s of active recovery at 35% V°O_2peak_, and 12 s of passive recovery. To simulate repeated sprint sequences commonly observed in team sports, participants completed an additional set of 5 × 4-s maximal sprints with 16 s of active recovery (35% V°O_2peak_) after the 8th and 16th sprint blocks in each half (31, 32). All sprint efforts were performed on an electronically braked cycle ergometer operating in Wingate mode (LEM module Wingate Test, Lode, Groningen, Netherlands) with the braking force set at 0.7 Nm·kg^−1^. The ergometer was equipped with toe clips to minimize foot slippage during maximal efforts (22, 25).

### Performance measurements

Peak power output (PPO) was defined as the highest power output achieved during each 8-s sprint (the primary sprint type), and mean power output (MPO) as the average power output over each 8-s sprint. PPO and MPO were calculated exclusively from the 8-s sprints (40 sprints in total; 20 per half). The 4-s sprints were not included in the calculation of PPO or MPO. Total work done was calculated as the sum of forty 8-s sprints and twenty 4-s sprints, excluding the active recovery periods. This calculation method was pre-specified prior to data analysis. Individual sprint values were averaged within each half to derive half-level mean values for PPO and MPO. The percentage increment and percentage decrement were calculated as:


$$\begin{array}{c} \text{Increment }(\%):\frac{\text{Caffeine-Placebo}}{\text{Placebo}}\times \text{100}\\ \text{Decrement }(\%):\frac{\text{First half-Second half}}{\text{First half}}\times \text{100} \end{array}$$

### Physiological measurements

Respiratory gas exchange was measured continuously breath-by-breath during the IST using a metabolic cart (Metalyzer 3B, Cortex, Leipzig, Germany). Oxygen uptake (V°O_2_), carbon dioxide production (V°CO_2_), respiratory exchange ratio, minute ventilation, tidal volume, respiratory frequency, end-tidal CO_2_ and O_2_ pressures, and ventilatory equivalents for O_2_ and CO_2_ were computed. The system was calibrated immediately before each trial with a precision gas mixture (15% O_2_ and 5% CO_2_) and a 3-L syringe for the flow meter. Errant data points due to coughing or swallowing were initially removed and thereafter any breath further than four standard deviations from the local mean was removed (33). Because cardiorespiratory responses exhibit a temporal delay, peaks in cardiorespiratory variables typically occurred several seconds after each 8-s sprint. To account for this delay, a 5-breath rolling average was applied, and representative sprint values were calculated by averaging the identified peak or nadir value with the adjacent data points immediately before and after (33). To ensure that recovery-phase data were not influenced by delayed physiological peak responses from the preceding sprint, cardiorespiratory and metabolic variables during recovery were averaged exclusively over the final 50 s of each active recovery interval. The same data processing procedures were applied to heart rate and SpO_2_ data. The onset of each sprint was marked by a digital trigger generated by the Lode Ergometry Manager Software (Lode B.V., Groningen, Netherlands), which synchronized ergometer power output with the breath-by-breath gas analysis system via a hardware connection.

Blood lactate was analyzed from samples collected before and after 10 min of gum chewing, as well as immediately after each IST half, using a lactate analyzer (Biosen, Cline, Germany). Ratings of overall perceived exertion (Borg 6–20 scale), breathing difficulty, and lower-limb heaviness (Borg CR10 scale) were recorded after every fifth sprint (34, 35). Heart rate (HR) and peripheral oxygen saturation (SpO_2_) were continuously monitored using a heart rate monitor (Vantage NV, POLAR, Kempele, Finland) and a fingertip pulse oximeter (Nellcor PM10N, Medtronic, MN, USA), respectively.

### Near-infrared spectroscopy measurements

Subjects were instrumented with two pairs of near-infrared spectroscopy (NIRS) probes to monitor absorption of light across cerebral and muscle tissue (Portalite, Artinis Medical Systems BV, Zetten, The Netherlands). The theoretical background, limitations, and measurement reliability of this device during exercise have been described in detail elsewhere (36, 37). One NIRS emitter and detector pair was placed over the left prefrontal lobe, between Fp1 and F3, and placement was further adjusted (< 5 mm) to obtain strong signal strength on every subject (9). Spacing between optodes was fixed at 5 cm using a black, plastic spacer held in place via double sided, stick disks and a black, tensioning headband to reduce the intrusion of extraneous light and the loss of transmitted NIR light from the field of investigation (38). A second NIRS emitter and detector pair was fixed on the distal part of the left vastus lateralis muscle belly (approximately 15 cm above the proximal border of the patella) using a black, plastic spacer with optode distance of 4.5 cm (9). Skinfold thickness was measured between the emitter and detector using a skinfold caliper (Harpenden Skinfold Caliper, Harpenden Ltd, UK) to account for the skin and adipose tissue thickness covering the muscle. The skinfold thickness (9.7 ± 5.2 mm) was less than half the distance between the emitter and the detector in each case. An indelible pen was used and pictures were taken to mark the position of the optode for subsequent visits.

A modified form of the Beer–Lambert Law was used to calculate micromolar changes in tissue oxyhemoglobin [O_2_Hb] and deoxyhemoglobin [HHb] across time using received optical densities from two continuous wavelengths of NIR light (763 and 855 nm) (39). An age-dependent differential optical pathlength factor for cerebral cortex (40) and of 4.95 for muscle (41) were used in this study. Data was acquired at 10 Hz and exported to Excel for analysis. Cerebral NIRS signals [(O_2_Hb), (HHb), and (THb)] were expressed as changes relative to a 2-min resting baseline, during which participants sat quietly on the ergometer before the warm-up. For muscle NIRS variables, these data were expressed as a percentage so that 0% represented a 5-s average immediately prior to the occlusion and 100% represented the maximum 5 s average. Occlusion was performed 3–5 min after cessation of the sprints while participants were positioned supine on an examination bed. Participants were instructed to place their feet flat on the examination bed with approximately 90° of knee flexion. A pneumatic tourniquet (BXZNJY-01 Thigh 5 × 120 cm, YIJIAYUNAN, China) was then placed around the proximal portion of the dominant thigh and inflated to 300 mmHg. Occlusion was maintained for 3–5 min or until the muscle deoxygenation signal plateaued (42). During the trials, markers were placed in the NIRS software (OxySoft) (Artinis Medical Systems B.V., Elst, Netherlands) at sprint onset to demarcate the 120-s sprint/recovery windows for analysis. A 10th-order zero-phase Butterworth low-pass filter with a cutoff frequency of 0.2 Hz was applied to remove motion artifacts and smooth pedaling-induced fluctuations, and the filtered signals were used for subsequent peak and nadir detection and analysis (42). Filtering was performed in the R environment (version 4.4.1; R Core Team, Vienna, Austria). Signal quality was visually inspected for each trial, and no trials were excluded due to motion artifacts or poor signal quality. A rolling approach was used to identify peak and nadir values within each individual sprint-recovery cycle (33). Peak [HHb] was identified during the final 5 s of each 8-s sprint phase, whereas nadir [HHb] was identified during the final 5 s of the subsequent recovery phase (42). The identified peak and nadir values from each valid sprint–recovery cycle were then averaged within each half of the IST to obtain half-specific outcomes. Total hemoglobin [THb] was derived as the sum of [O_2_Hb] and [HHb] and used as an index of changes in regional blood volume (37). Reoxygenation capacity (ΔReoxy (%) = HHb_peak_ – HHb_nadir_) was calculated as the difference from peak [HHb] (sprint) to nadir [HHb] (recovery) in the vastus lateralis and used as a parameter of the quality of vastus lateralis metabolic recovery between sprints (43).

### Statistical analyses

Data was analyzed R (version 4.4.1; R Core Team, Vienna, Austria). Date was reported as mean ± standard deviation (SD), with statistical significance set at *p* < 0.05. Data normality was assessed using the Shapiro-Wilk test and visual inspection of Q-Q plots. A two-way repeated-measures analysis of variance (ANOVA) [time (first vs. second half) × condition (placebo, caffeine)] was used to analyze total work done, PPO, MPO, SpO_2_, cardiorespiratory variables, cerebral oxygenation parameters [(O_2_Hb), (HHb) and (THb)], and muscle oxygenation parameters [(O_2_Hb), (HHb), (THb) and ΔReoxy]. Similarly, two-way repeated-measures ANOVA [time (sprint number: 5, 10, …, 40) or (testing points: pre, post, first half, second half) × condition (placebo, caffeine)] was used to analyze perceptual ratings (overall perceived exertion, lower-limb discomfort, breathing difficulty) and blood lactate. When Mauchly's test indicated a violation of sphericity, the Greenhouse-Geisser correction was applied to adjust the degrees of freedom (epsilon values ranged from 0.357 to 0.354). Where significant main or interaction effects were found, Bonferroni-corrected *post-hoc* analyses were performed. Effect sizes were reported as partial eta-squared (η*p*^2^) for main effects. For pairwise *post-hoc* comparisons, Hedges'*g*_*z*_, a paired standardized mean-difference estimator, was calculated to account for the crossover design. Hedges'*g*_*z*_ was calculated as (44):


$$\begin{array}{l} g_{z}=J\times \frac{\bar{D}}{SD_{D}} \end{array}$$

with the small-sample bias correction factor and confidence interval calculated as (44):


$$\begin{array}{l} J=1-\frac{3}{4\;\left(n-1\right)-1}\\ 95\%\;CI\;=\;g_{z\;}\pm \;t_{\alpha /2}\times SE_{g}\\ SE_{g}=\sqrt{\frac{1}{n}\;+\;\frac{g_{z}^{2}}{2n}} \end{array}$$

where $\bar{D}$ represents the mean paired difference between caffeine and placebo conditions, *SD*_*D*_ represents the standard deviation of paired differences, *J* represents the small-sample bias correction factor, *n* represents the number of participants, and *t*_α/2_ represents the two-tailed critical value from the *t*-distribution with *n* – 1 degrees of freedom. Mean paired differences with corresponding 95% confidence intervals were additionally reported for all pairwise comparisons. Given the number of outcomes analyzed, we prespecified total work done as the primary outcome and peak and mean power output as secondary outcomes. All other measures (cardiorespiratory, perceptual, blood lactate, and cerebral and muscle oxygenation variables) were considered exploratory outcomes.

## Results

### Prolonged intermittent-sprint performance

#### Total work done

Significant main effects of condition [*F*_(1, 9)_ = 15.97, *p* = 0.003, η*p*^2^ = 0.640] and time [*F*_(1, 9)_ = 40.58, *p* < 0.001, η*p*^2^ = 0.818] were observed for total work (Figure 1A). *Post hoc* analysis revealed that caffeine increased total work done compared with placebo in both the first [143 ± 28 vs. 137 ± 25 kJ; mean difference 6 kJ, 95% CI (2.71, 10.08), *g*_*z*_ = 1.14, 95% CI (0.34, 1.93); *p* = 0.003] and second half [131 ± 24 vs. 127 ± 22 kJ, mean difference 4 kJ, 95% CI (1.18, 6.57), *g*_*z*_ = 0.94, 95% CI (0.20, 1.69); *p* = 0.009], with no significant difference in percent increase between halves [4.4% vs. 2.9%, mean difference 1.5%, 95% CI (−0.44, 3.38), *g*_*z*_ = 0.50, 95% CI (−0.15, 1.16); *p* = 0.116; Figure 1A–B]. Furthermore, total work done declined from the first to the second half in both caffeine [143 ± 28 vs. 131 ± 24, mean difference 12 kJ, 95% CI (−17.50, −7.90), *g*_*z*_ = 1.73, 95% CI (−2.71, −0.75); *p* < 0.001] and placebo conditions [137 ± 25 vs. 127 ± 22, mean difference 10 kJ, 95% CI (−13.90, −6.45), *g*_*z*_ = 1.79, 95% CI (−2.79, −0.79); *p* < 0.001], with no significant difference in the percentage decrement between conditions [8.6% vs. 7.3%, mean difference 1.3%, 95% CI (−0.41, 2.95), *g*_*z*_ = 0.50, 95% CI (−0.15, 1.16); *p* = 0.12; Figure 1C].

**Figure 1:**
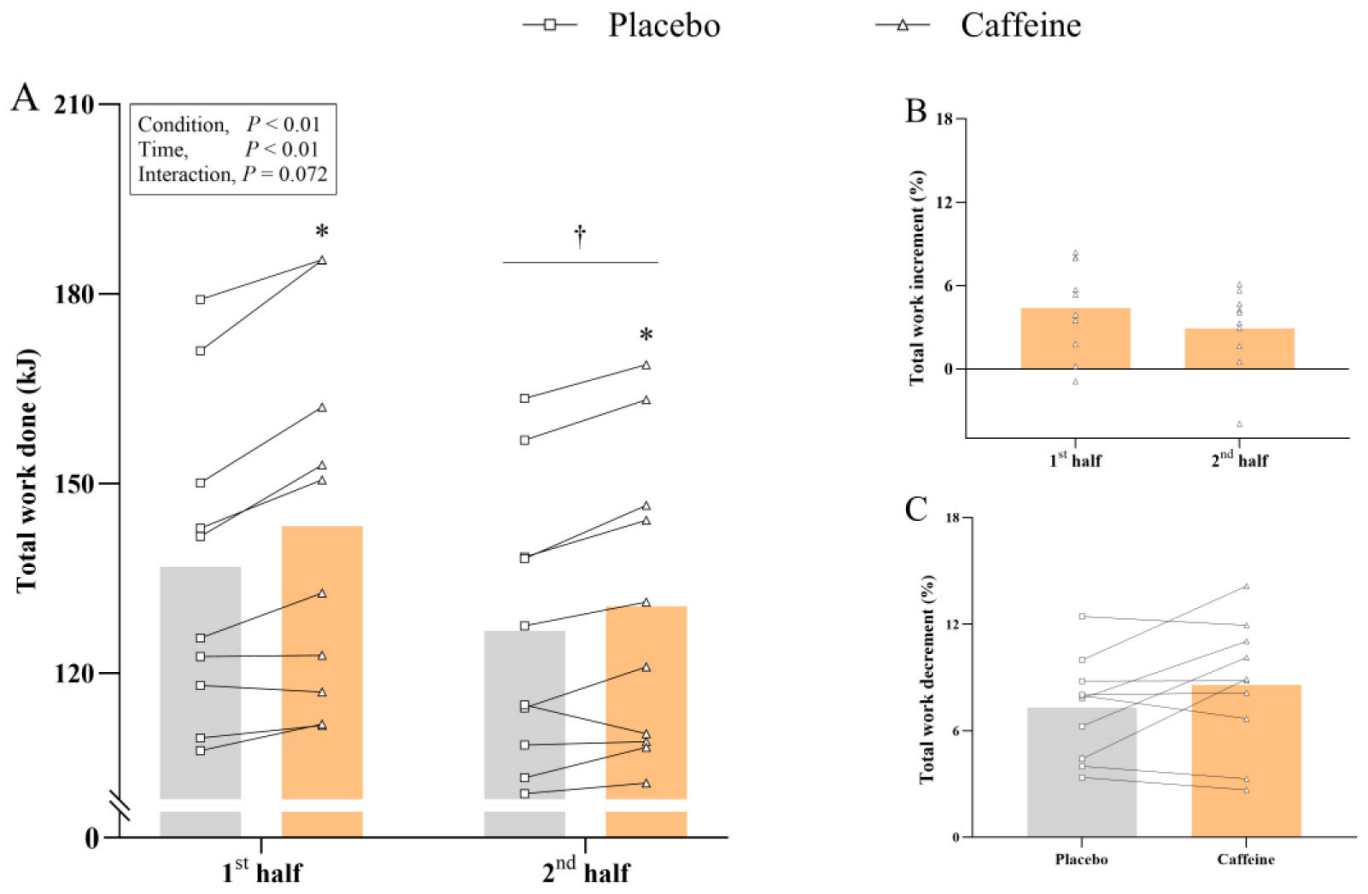
Total work dones **(A)**, percentage increment **(B)**, and percent decrement **(C)** during the intermittent sprint test with caffeine ingestion. Data are presented as individual paired observations, with each participant's values connected by a line. Error bars represent SD. *p < 0.05 vs. placebo,^†^*p* < 0.05 vs. first half.

#### Power output

Significant main effects of condition [*F*_(1, 9)_ = 23.67, *p* < 0.001, η*p*^2^ = 0.724] and time [*F*_(1, 9)_ = 5.14, *p* = 0.049, η*p*^2^ = 0.364] were observed for PPO (Figure 2A). *Post hoc* analysis revealed that caffeine increased PPO compared with placebo in both the first [1,015 ± 209 vs. 976 ± 207 W, mean difference 39 W, 95% CI (24.16, 54.62), *g*_*z*_ = 1.69, 95% CI (0.73, 2.66); *p* < 0.001] and second half [977 ± 207 vs. 948 ± 190 W, mean difference 29 W, 95% CI (9.18, 50.20), *g*_*z*_ = 0.95, 95% CI (0.20, 1.69); *p* = 0.009; Figure 2A].

**Figure 2:**
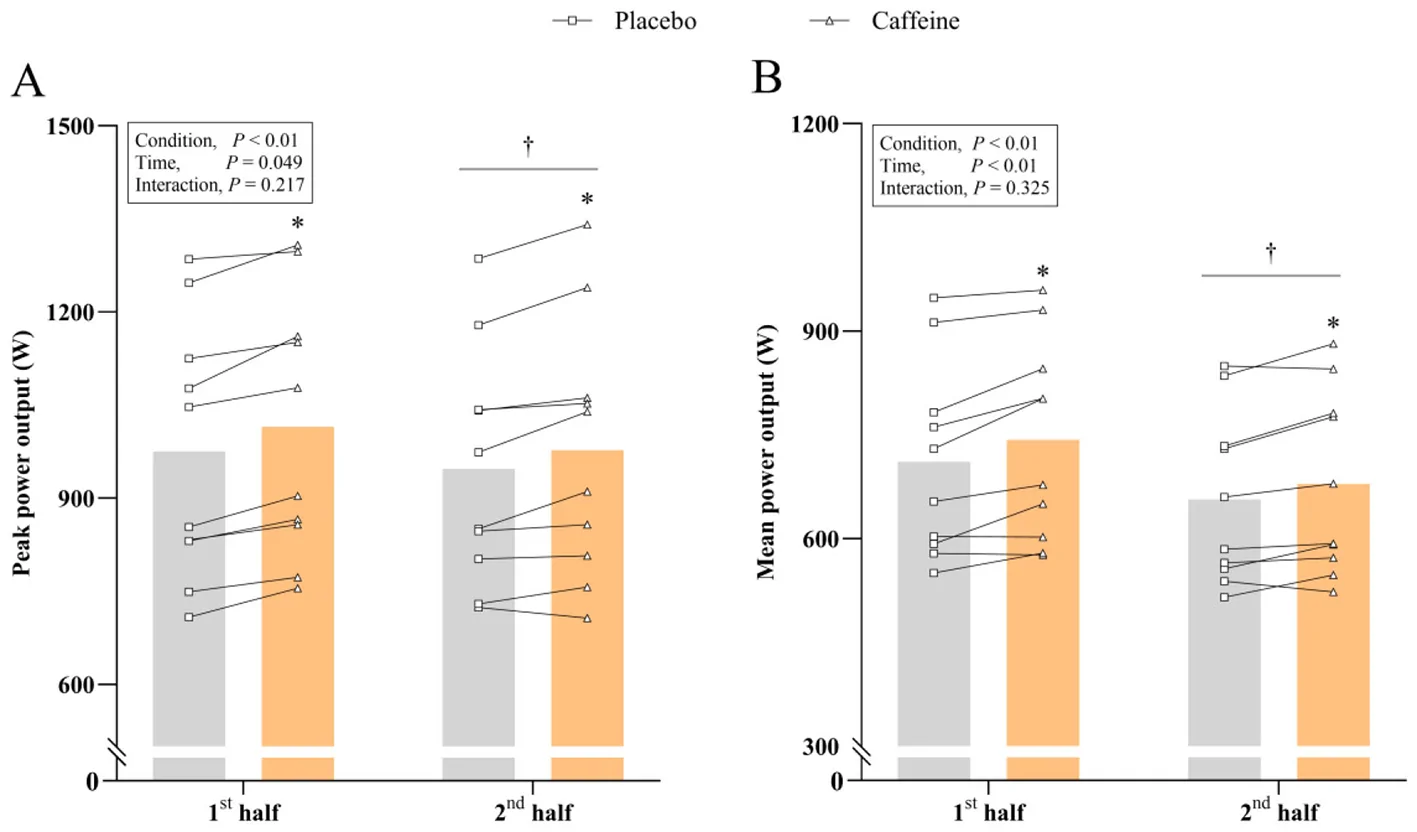
Peak power output **(A)** and Mean power output **(B)** during the intermittent sprint test with caffeine ingestion. Data are presented as individual paired observations, with each participant's values connected by a line. Error bars represent SD. *p < 0.05 vs. placebo,^†^*p* < 0.05 vs. first half.

A significant main effect of condition [*F*_(1, 9)_ = 17.44 *p* = 0.002, η*p*^2^ = 0.660] and time [*F*_(1, 9)_ = 42.77, *p* < 0.001, η*p*^2^ = 0.826] was found for MPO (Figure 2B). *Post-hoc* tests showed that caffeine increased MPO compared with placebo in both the first [743 ± 144 vs. 711 ± 140 W, mean difference 32 W, 95% CI (12.48, 50.30), *g*_*z*_ = 1.09, 95% CI (0.30, 1.86); *p* = 0.004] and second half [679 ± 132 vs. 657 ± 124 W, mean difference 22 W, 95% CI (6.16, 38.42), *g*_*z*_ = 0.90, 95% CI (0.17, 1.64); *p* = 0.012; Figure 2B].

### NIRS measurements

### Cerebral analysis

A significant main effect of condition was observed for cerebral [O_2_Hb] [*F*_(1, 9)_ = 12.25, *p* = 0.007, η*p*^2^ = 0.576] and [THb] [*F*_(1, 9)_ = 17.63, *p* = 0.002, η*p*^2^ = 0.662; Table 1]. *Post hoc* analyses showed that compared with placebo, cerebral [O_2_Hb] increased with caffeine in the first half [18.5 ± 9.0 vs. 14.6 ± 6.8 μM, mean difference 3.9 μM, 95% CI (0.71, 6.94), *g*_*z*_ = 0.80, 95% CI (0.09, 1.51); *p* = 0.020] and the second half [18.1 ± 8.9 vs. 12.9 ± 6.0 μM, mean difference 5.2 μM, 95% CI (1.84, 8.55), *g*_*z*_ = 1.01, 95% CI (0.25, 1.77); *p* = 0.006]. Furthermore, compared with placebo, caffeine increased cerebral [THb] during both the first half [24.5 ± 5.3 vs. 17.6 ± 6.0 μM, mean difference 6.9 μM, 95% CI (2.01, 11.87), *g*_*z*_ = 0.92, 95% CI (0.18, 1.66); *p* = 0.011] and the second half [23.5 ± 8.4 vs. 16.5 ± 5.4 μM, mean difference 7 μM, 95% CI (2.62, 11.48), *g*_*z*_ = 1.04, 95% CI (0.27, 1.81); *p* = 0.005; Table 1].

**Table 1:** Muscle and cerebral oxygenation responses during the repeated-sprint protocol under placebo and caffeine conditions.

Variable	Half	Placebo	Caffeine	Repeated-measures ANOVA								
				**Condition**			**Half**			**Condition** × **Half**		
				*F*_(df1, df2)_	***p***	η*p*^2^	*F*_(df1, df2)_	***p***	η*p*^2^	*F*_(df1, df2)_	***p***	η*p*^2^
Cerebral Δ[THb] (μM)	1st half	17.6 ± 6.0	24.5 ± 5.3^*^	*F*_(1, 9)_ = 17.63	**0.002**	0.662	*F*_(1, 9)_ = 0.25	0.631	0.027	*F*_(1, 9)_ = 0.00	0.968	0.000
	2nd half	16.5 ± 5.3	23.5 ± 8.4^*^									
Cerebral Δ[O_2_Hb] (μM)	1st half	14.6 ± 6.8	18.5 ± 9.0^*^	*F*_(1, 9)_ = 12.25	**0.007**	0.576	*F*_(1, 9)_ = 0.28	0.610	0.030	*F*_(1, 9)_ = 1.21	0.300	0.118
	2nd half	12.9 ± 6.0	18.1 ± 8.9^*^									
Cerebral Δ[HHb] (μM)	1st half	3.9 ± 1.0	4.6 ± 1.7	*F*_(1, 9)_ = 0.84	0.383	0.086	*F*_(1, 9)_ = 2.16	0.175	0.194	*F*_(1, 9)_ = 0.02	0.887	0.002
	2nd half	4.3 ± 1.4	5.1 ± 2.4									
Muscle Δ[HHb] (%)	1st half	20.6 ± 17.2	37.8 ± 13.0^*^	*F*_(1, 9)_ = 11.82	**0.007**	0.568	*F*_(1, 9)_ = 2.18	0.174	0.195	*F*_(1, 9)_ = 1.08	0.326	0.107
	2nd half	36.5 ± 25.7	41.5 ± 32.5									
Muscle Δ[O_2_Hb] (%)	1st half	42.7 ± 7.2	43.9 ± 14.9	*F*_(1, 9)_ = 0.01	0.924	0.001	*F*_(1, 9)_ = 9.34	**0.014**	0.509	*F*_(1, 9)_ = 0.77	0.404	0.078
	2nd half	54.1 ± 8.6^†^	51.9 ± 12.3									
Muscle Δ[THb] (%)	1st half	45.9 ± 12.9	48.2 ± 24.3	*F*_(1, 9)_ = 0.09	0.774	0.010	*F*_(1, 9)_ = 13.99	**0.005**	0.609	*F*_(1, 9)_ = 0.01	0.939	0.001
	2nd half	58.7 ± 11.5^†^	60.1 ± 11.6									
Muscle ΔReoxy (%)	1st half	47.9 ± 12.2	68.0 ± 15.3^*^	*F*_(1, 9)_ = 7.95	**0.023**	0.498	*F*_(1, 9)_ = 20.23	**0.002**	0.717	*F*_(1, 9)_ = 2.97	0.123	0.271
	2nd half	34.5 ± 20.3^†^	39.4 ± 16.6^†^									

Bold values indicate statistical significance. Data are means ± SD. THb, total hemoglobin; O_2_Hb, oxy-hemoglobin; HHb, deoxy-hemoglobin; ΔReoxy, magnitude of muscle reoxygenation response; ANOVA, analysis of variance; F, F-statistic from repeated-measures ANOVA; ηp^2^, partial eta squared; SD, standard deviation.

* *p* < 0.05 vs. placebo;^†^*p* < 0.05 vs. first half.

### Muscle analysis

There were significant main effects of condition on muscle [HHb] [*F*_(1, 9)_ = 11.82, *p* = 0.007, η*p*^2^ = 0.568] and ΔReoxy [*F*_(1, 9)_ = 7.95, *p* = 0.023, η*p*^2^ = 0.498; Table 1]. *Post hoc* tests showed that compared with placebo, caffeine increased muscle [HHb] and ΔReoxy during the first half [(HHb): 37.8 ± 13.0 vs. 20.6 ± 17.2 %, mean difference 17.2%, 95% CI (5.12, 29.29), *g*_*z*_ = 0.93, 95% CI (0.19, 1.67); *p* = 0.010; ΔReoxy: 68.0 ± 15.3 vs. 48.0 ± 12.2%, mean difference 20%, 95% CI (7.62, 32.43), *g*_*z*_ = 1.12, 95% CI (0.29, 1.95); *p* = 0.005, *g* = 1.12]. However, in the second half, caffeine did not significantly increase muscle [HHb] [41.5 ± 32.5 vs. 36.5 ± 25.7%, mean difference 5%, 95% CI (−12.64, 22.71), *g*_*z*_ = 0.18, 95% CI (−0.44, 0.81); *p* = 0.535] or ΔReoxy [39.4 ± 16.6 vs. 34.5 ± 20.3%, mean difference 4.9%, 95% CI (−11.19, 21.00), *g*_*z*_ = 0.21, 95% CI (−0.45, 0.87); *p* = 0.502] compared with placebo (Table 1).

### Blood lactate concentration

Significant main effects were observed for condition [*F*_(1, 9)_ = 6.10, *p* = 0.036, η*p*^2^ = 0.404] and time [*F*_(1.38, 12.45)_ = 90.08, *p* < 0.001, η*p*^2^ = 0.909; Figure 3]. *Post hoc* analyses showed that blood lactate concentrations were higher following both the first [13.5 ± 5.0 vs. 10.0 ± 2.9 mmol/L, mean difference 3.5 mmol/L, 95% CI (0.03, 6.91), *g* = 0.66, 95% CI (−0.03, 1.34); *p* = 0.048] and second halves [10.1 ± 3.4 vs. 7.8 ± 2.5 mmol/L, mean difference 2.3 mmol/L, 95% CI (0.03, 6.91), *g* = 0.70, 95% CI (0.01, 1.39); *p* = 0.038] with caffeine compared with placebo.

**Figure 3:**
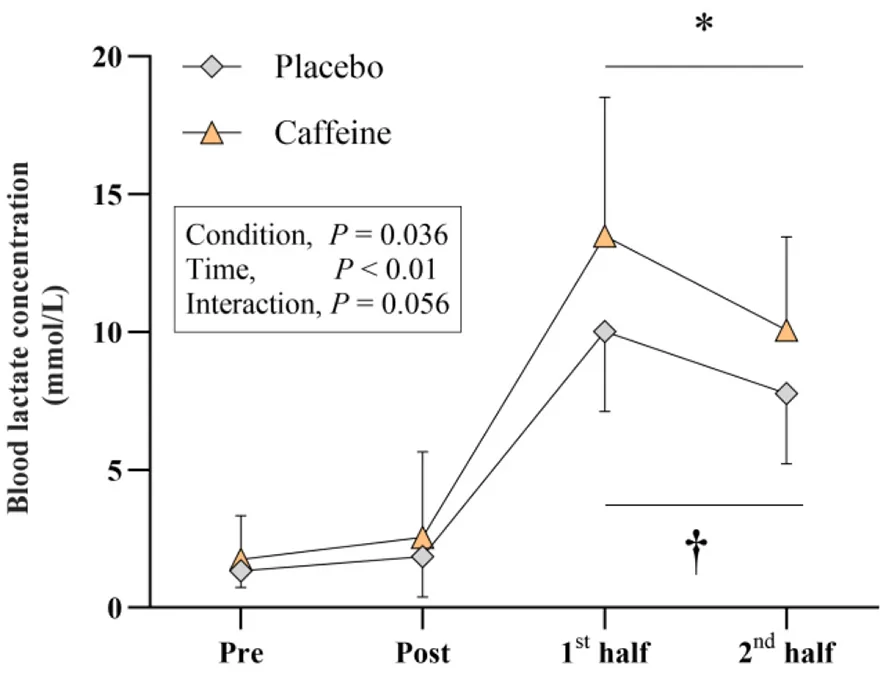
Blood lactate concentration during intermittent sprint test with caffeine ingestion. *p < 0.05 vs. placebo,^†^*p* < 0.05 vs. baseline (Pre).

### Perceptual and physiological responses

No significant main effects of condition were observed for overall perceived exertion [*F*_(1, 9)_ = 0.07, *p* = 0.800, η*p*^2^ = 0.008], breathing difficulty [*F*_(1, 9)_ = 0.10, *p* = 0.760, η*p*^2^ = 0.012], or lower-limb discomfort [*F*_(1, 9)_ = 0.06, *p* = 0.810, η*p*^2^ = 0.007; Figure 4].

**Figure 4:**
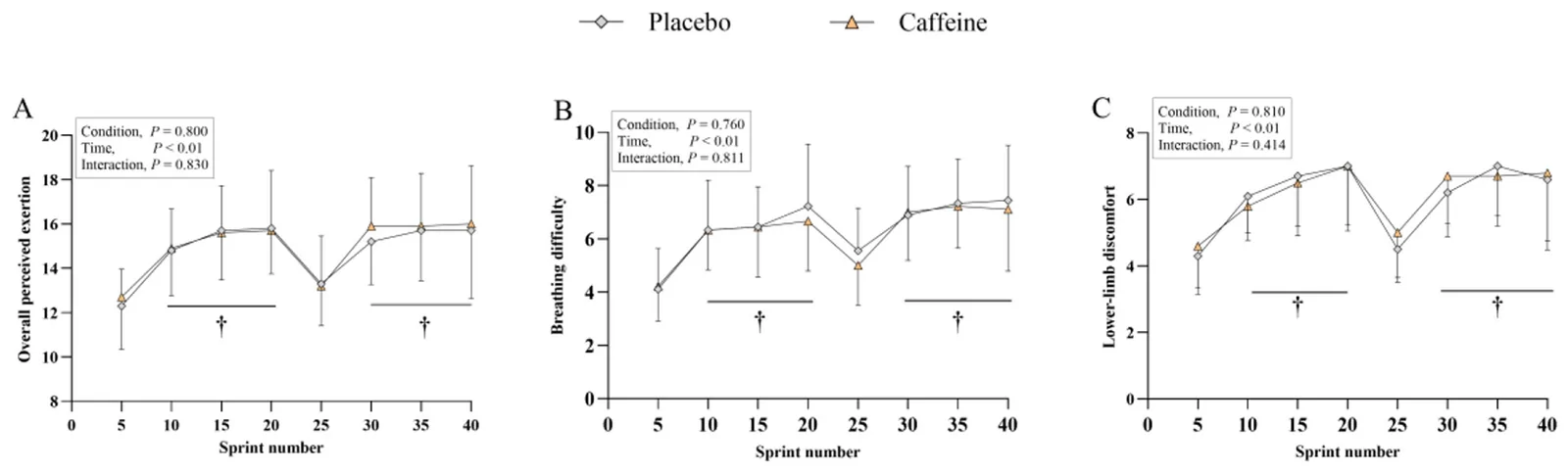
Ratings of perceived exertion related to overall exertion **(A)**, breathing difficulty **(B)**, and lower-limb heaviness **(C)** during intermittent sprint test with caffeine ingestion.^†^*p* < 0.05 vs. sprint number 5.

During the sprint period of the IST, there were significant main effects of condition on heart rate [*F*_(1, 9)_ = 8.08, *p* = 0.019, η*p*^2^ = 0.473], minute ventilation [*F*_(1, 9)_ = 16.12, *p* = 0.003, η*p*^2^ = 0.642], tidal volume [*F*_(1, 9)_ = 12.54, *p* = 0.006, η*p*^2^ = 0.582], ventilatory equivalents for O_2_ [*F*_(1, 9)_ = 65.80, *p* < 0.001, η*p*^2^ = 0.879] and CO_2_ [*F*_(1, 9)_ = 27.71, *p* < 0.001, η*p*^2^ = 0.755], and end-tidal O_2_ [*F*_(1, 9)_ = 99.41, *p* < 0.001, η*p*^2^ = 0.917] and CO_2_ [*F*_(1, 9)_ = 17.24, *p* = 0.002, η*p*^2^ = 0.657] partial pressures (Table 2). *Post-hoc* analyses showed that compared with placebo, caffeine increased minute ventilation [112 ± 26 vs. 105 ± 25 L/min, mean difference 7 L/min, 95% CI (4.55, 9.93), *g*_*z*_ = 1.76, 95% CI (0.77, 2.75); *p* < 0.001] and ventilatory equivalents for O_2_ [41.6 ± 5.1 vs. 37.9 ± 4.5, mean difference 3.7, 95% CI (2.50, 4.93), *g*_*z*_ = 2.00, 95% CI (0.93, 3.08); *p* < 0.001] during the first half, whereas these variables [minute ventilation: 103 ± 25 vs. 101 ± 25 L/min, mean difference 2 L/min, 95% CI (−2.06, 6.31), *g*_*z*_ = 0.33, 95% CI (−0.30, 0.97); *p* = 0.281]; ventilatory equivalents for O_2_ [37.9 ± 4.1 vs. 37.2 ± 4.3, mean difference 0.7, 95% CI (−0.08, 1.49), *g*_*z*_ = 0.58, 95% CI (−0.09, 1.25); *p* = 0.074] were not elevated during the second half. In addition, compared with placebo, caffeine increased heart rate [first half: 175 ± 8 vs. 169 ± 6 beats/min, mean difference 6 beats/min, 95% CI (0.20, 11.93), *g*_*z*_ = 0.68, 95% CI (0.01, 1.36); *p* = 0.044; second half: 175 ± 6 vs. 169 ± 5 beats/min, mean difference 6 beats/min, 95% CI (2.17, 10.82), *g*_*z*_ = 0.98, 95% CI (0.23, 1.74); *p* = 0.007], tidal volume [first half: 2.35 ± 0.65 vs. 2.24 ± 0.61 L, mean difference 0.11 L, 95% CI (0.04, 0.19), *g*_*z*_ = 0.95, 95% CI (0.21, 1.70); *p* = 0.009; second half: 2.21 ± 0.60 vs. 2.15 ± 0.56 L, mean difference 0.06 L, 95% CI (0.01, 0.11), *g*_*z*_ = 0.70, 95% CI (0.01, 1.39); *p* = 0.038], ventilatory equivalents for CO_2_ [first half: 39.4 ± 3.6 vs. 36.7 ± 4.3, mean difference 2.7, 95% CI (1.22, 4.35), *g* = 1.16, 95% CI (0.36, 1.96); *p* = 0.003; second half: 39.7 ± 3.6 vs. 37.4 ± 4.5, mean difference 2.3, 95% CI (1.31, 3.33), *g*_*z*_ = 1.50, 95% CI (0.59, 2.40); *p* < 0.001], and end-tidal O_2_ partial pressure [first half: 97.2 ± 2.4 vs. 94.6 ± 2.4 mmHg, mean difference 2.6 mmHg, 95% CI (1.92, 3.26), *g*_*z*_ = 2.52, 95% CI (1.25, 3.79); *p* < 0.01; second half: 95.2 ± 3.8 vs. 93.4 ± 3.3 mmHg, mean difference 1.8 mmHg, 95% CI (0.69, 2.88), *g*_*z*_ = 1.06, 95% CI (0.29, 1.84); *p* < 0.01] across both halves, while reducing end-tidal CO_2_ partial pressure (first half: 24.1 ± 2.0 vs. 25.1 ± 2.4 mmHg, mean difference −1 mmHg, 95% CI (−1.55, −0.44), *g*_*z*_ = −1.17, 95% CI (−1.98, −0.36); *p* < 0.001; second half: 24.8 ± 2.8 vs. 25.9 ± 3.2 mmHg, mean difference −1.1 mmHg, 95% CI (−1.88, −0.41), *g*_*z*_ = −1.02, 95% CI (−1.79, −0.25); *p* = 0.005).

**Table 2:** Cardiorespiratory variables during intermittent sprint under placebo and caffeine conditions.

**Variable**	Half	Placebo	Caffeine	Repeated-measures ANOVA								
				**Condition**			**Half**			**Condition** × **Half**		
				*F*_(df1, df2)_	***p***	η*p*^2^	*F*_(df1, df2)_	***p***	η*p*^2^	*F*_(df1, df2)_	***p***	η*p*^2^
O_2_ uptake (L/min)	1st half	2.81 ± 0.30	2.84 ± 0.30	*F*_(1, 9)_ = 2.22	0.171	0.198	*F*_(1, 9)_ = 3.59	0.091	0.285	*F*_(1, 9)_ = 0.17	0.687	0.019
	2nd half	2.86 ± 0.39	2.91 ± 0.35									
CO_2_ elimination (L/min)	1st half	2.73 ± 0.32	2.77 ± 0.32	*F*_(1, 9)_ = 4.03	0.076	0.309	*F*_(1, 9)_ = 4.50	0.063	0.333	*F*_(1, 9)_ = 0.13	0.727	0.014
	2nd half	2.62 ± 0.32	2.66 ± 0.28									
Minute ventilation (L/min)	1st half	105 ± 25	112 ± 26^*^	*F*_(1, 9)_ = 16.12	**0.003**	0.642	*F*_(1, 9)_ = 6.32	**0.033**	0.413	*F*_(1, 9)_ = 6.19	**0.035**	0.407
	2nd half	101 ± 25	103 ± 25^†^									
Tidal volume (L)	1st half	2.24 ± 0.61	2.35 ± 0.65^*^	*F*_(1, 9)_ = 12.54	**0.006**	0.582	*F*_(1, 9)_ = 12.19	**0.007**	0.575	*F*_(1, 9)_ = 2.30	0.163	0.204
	2nd half	2.15 ± 0.56^†^	2.21 ± 0.60^†^									
Breathing frequency (breaths/min)	1st half	68 ± 15	69 ± 15	*F*_(1, 9)_ = 0.06	0.805	0.007	*F*_(1, 9)_ = 4.09	0.074	0.313	*F*_(1, 9)_ = 1.00	0.343	0.100
	2nd half	72 ± 16	71 ± 15									
Respiratory exchange ratio	1st half	1.06 ± 0.06	1.06 ± 0.06	*F*_(1, 9)_ = 0.27	0.616	0.029	*F*_(1, 9)_ = 117.43	**<** **0.001**	0.929	*F*_(1, 9)_ = 5.54	**0.043**	0.381
	2nd half	0.96 ± 0.06^†^	0.98 ± 0.06^†^									
Ventilatory equivalent for O_2_	1st half	37.9 ± 4.5	41.6 ± 5.1^*^	*F*_(1, 9)_ = 65.80	**<** **0.001**	0.880	*F*_(1, 9)_ = 7.21	**0.025**	0.445	*F*_(1, 9)_ = 17.45	**0.002**	0.660
	2nd half	37.2 ± 4.3	37.9 ± 4.1^†^									
Ventilatory equivalent for CO_2_	1st half	36.7 ± 4.3	39.4 ± 3.6^*^	*F*_(1, 9)_ = 27.71	**<** **0.001**	0.755	*F*_(1, 9)_ = 0.95	0.355	0.096	*F*_(1, 9)_ = 0.51	0.493	0.054
	2nd half	37.4 ± 4.5	39.7 ± 3.6^*^									
End-tidal O_2_ partial pressure (mmHg)	1st half	94.6 ± 2.4	97.2 ± 2.4^*^	*F*_(1, 9)_ = 99.41	**<** **0.001**	0.917	*F*_(1, 9)_ = 3.95	0.078	0.305	*F*_(1, 9)_ = 1.41	0.265	0.135
	2nd half	93.4 ± 3.3	95.2 ± 3.8^*^									
End-tidal CO_2_ partial pressure (mmHg)	1st half	25.1 ± 2.4	24.1 ± 2.0^*^	*F*_(1, 9)_ = 17.24	**0.002**	0.657	*F*_(1, 9)_ = 2.92	0.122	0.245	*F*_(1, 9)_ = 0.36	0.563	0.038
	2nd half	25.9 ± 3.2	24.8 ± 2.8^*^									
Heart rate (beats/min)	1st half	169 ± 6	175 ± 8^*^	*F*_(1, 9)_ = 8.08	**0.019**	0.473	*F*_(1, 9)_ = 0.01	0.931	0.001	*F*_(1, 9)_ = 0.15	0.710	0.016
	2nd half	169 ± 5	175 ± 6 ^*^									
SpO_2_ (%)	1st half	92 ± 1	93 ± 1	*F*_(1, 9)_ = 3.28	0.103	0.267	*F*_(1, 9)_ = 2.33	0.162	0.205	*F*_(1, 9)_ = 0.08	0.786	0.009
	2nd half	92 ± 1	93 ± 1									

Bold values indicate statistical significance. Data are means ± SD. O_2_, oxygen; CO_2_, carbon dioxide; SpO_2_, peripheral oxygen saturation; ANOVA, analysis of variance; F, F-statistic from repeated-measures ANOVA; ηp^2^, partial eta squared; SD, standard deviation.

* *p* < 0.05 vs. placebo;^†^*p* < 0.05 vs. first half.

During the recovery period of the IST, a significant main effect of condition was observed for O_2_ uptake [*F*_(1, 9)_ = 5.30, *p* = 0.047, η*p*^2^ = 0.370], CO_2_ elimination [*F*_(1, 9)_ = 7.83, *p* = 0.021, η*p*^2^ = 0.465], minute ventilation [*F*_(1, 9)_ = 22.05, *p* = 0.001, η*p*^2^ = 0.710], tidal volume [*F*_(1, 9)_ = 65.03, *p* < 0.001, η*p*^2^ = 0.878], ventilatory equivalents for O_2_ [*F*_(1, 9)_ = 26.05, *p* < 0.001, η*p*^2^ = 0.743] and CO_2_ [*F*_(1, 9)_ = 22.21, *p* = 0.001, η*p*^2^ = 0.712], and end-tidal O_2_ [*F*_(1, 9)_ = 11.58, *p* = 0.001, η*p*^2^ = 0.563] and CO_2_ partial pressures [*F*_(1, 9)_ = 19.56, *p* = 0.002, η*p*^2^ = 0.685; Table 3]. *Post-hoc* analyses showed that compared with placebo, caffeine increased O_2_ uptake during the first half (2.18 ± 0.30 vs. 2.08 ± 0.33 L/min, mean difference 0.1 L/min, 95% CI (0.01, 0.19), *g*_*z*_ = 0.69, 95% CI (0.01, 1.39); *p* = 0.039), whereas this response was not elevated during the second half (2.16 ± 0.27 vs. 2.08 ± 0.33 L/min, mean difference 0.08 L/min, 95% CI (−0.02, 0.19), *g*_*z*_ = 0.55, 95% CI (−0.11, 1.22); *p* = 0.087). In addition, compared with placebo, caffeine increased CO_2_ elimination (first half: 2.16 ± 0.26 vs. 2.05 ± 0.32 L/min, mean difference 0.11 L/min, 95% CI (0.01, 0.21), *g*_*z*_ = 0.75, 95% CI (0.05, 1.45); *p* = 0.030; second half: 2.01 ± 0.20 vs. 1.9 ± 0.29 L/min, mean difference 0.11 L/min, 95% CI (0.01, 0.21), *g*_*z*_ = 0.71, 95% CI (0.02, 1.40); *p* = 0.036), minute ventilation [first half: 77 ± 12 vs. 68 ± 13 L/min, mean difference 9 L/min, 95% CI (3.69, 15.07), *g*_*z*_ = 1.08, 95% CI (0.30, 1.86); *p* = 0.004; second half: 73 ± 10 vs. 63 ± 11 L/min, mean difference 10 L/min, 95% CI (5.36, 14.14), *g*_*z*_ = 1.45, 95% CI (0.56, 2.34); *p* < 0.001], tidal volume [first half: 2.10 ± 0.65 vs. 1.90 ± 0.64 L, mean difference 0.2 L/min, 95% CI (0.12, 0.28), *g*_*z*_ = 1.65, 95% CI (0.69, 2.60); *p* < 0.001; second half: 1.93 ± 0.57 vs. 1.78 ± 0.57 L, mean difference 0.15 L/min, 95% CI (0.10, 0.21), *g*_*z*_ = 1.71, 95% CI (0.74, 2.69); *p* < 0.001], ventilatory equivalents for O_2_ [first half: 33.0 ± 3.4 vs. 30.0 ± 3.0, mean difference 3, 95% CI (1.15, 4.93), *g*_*z*_ = 1.05, 95% CI (0.28, 1.82); *p* = 0.005; second half: 31.3 ± 3.2 vs. 28.0 ± 2.9, mean difference 3.3, 95% CI (1.71, 4.94), *g*_*z*_ = 1.34, 95% CI (0.49, 2.20); *p* = 0.001] and CO_2_ (first half: 33.2 ± 3.6 vs. 30.6 ± 3.0, mean difference 2.6, 95% CI (0.98, 4.23), *g*_*z*_ = 1.04, 95% CI (0.28, 1.82); *p* = 0.005; second half: 33.5 ± 3.0 vs. 30.4 ± 3.3, mean difference 3.1, 95% CI (1.61, 4.54), *g*_*z*_ = 1.37, 95% CI (0.51, 2.23); *p* = 0.001], and end-tidal O_2_ partial pressure across both halves (first half: 92.0 ± 2.3 vs. 90.2 ± 2.1 mmHg, mean difference 1.8 mmHg, 95% CI (0.03, 3.40), *g*_*z*_ = 0.67, 95% CI (0.01, 1.35); *p* = 0.046; second half: 90.6 ± 3.3 vs. 87.8 ± 2.9 mmHg, mean difference 2.8 mmHg, 95% CI (1.03, 4.59), *g*_*z*_ = 1.03, 95% CI (0.27, 1.80); *p* = 0.005], while reducing end-tidal CO_2_ partial pressure (first half: 27.2 ± 2.4 vs. 29.3 ± 2.7 mmHg, mean difference −2.1 mmHg, 95% CI (−3.34, −0.80), *g*_*z*_ = −1.07, 95% CI (−1.84, −0.29); *p* = 0.005; second half: 27.6 ± 2.3 vs. 30.3 ± 3.3 mmHg, mean difference −2.7 mmHg, 95% CI (−4.13, −1.16), *g*_*z*_ = −1.16, 95% CI (−1.97, −0.36); *p* = 0.002].

**Table 3:** Cardiorespiratory variables during the recovery period under placebo and caffeine conditions.

**Variable**	Half	Placebo	Caffeine	Repeated-measures ANOVA								
				**Condition**			**Half**			**Condition** × **Half**		
				*F*_(df1, df2)_	***p***	η*p*^2^	*F*_(df1, df2)_	***p***	η*p*^2^	*F*_(df1, df2)_	***p***	η*p*^2^
O_2_ uptake (L/min)	1st half	2.08 ± 0.33	2.18 ± 0.30^*^	*F*_(1, 9)_ = 5.30	**0.047**	0.370	*F*_(1, 9)_ = 0.09	0.766	0.010	*F*_(1, 9)_ = 0.26	0.622	0.028
	2nd half	2.08 ± 0.33	2.16 ± 0.27									
CO_2_ elimination (L/min)	1st half	2.05 ± 0.32	2.16 ± 0.26^*^	*F*_(1, 9)_ = 7.83	**0.021**	0.465	*F*_(1, 9)_ = 44.67	**<** **0.001**	0.832	*F*_(1, 9)_ = 0.00	0.949	0.000
	2nd half	1.90 ± 0.29^†^	2.01 ± 0.20^†^									
Minute ventilation (L/min)	1st half	68 ± 13	77 ± 12^*^	*F*_(1, 9)_ = 22.05	**0.001**	0.710	*F*_(1, 9)_ = 7.55	**0.023**	0.456	*F*_(1, 9)_ = 0.04	0.848	0.004
	2nd half	63 ± 11^†^	73 ± 10^*^									
Tidal volume (L)	1st half	1.90 ± 0.64	2.10 ± 0.65^*^	*F*_(1, 9)_ = 65.03	**<** **0.001**	0.878	*F*_(1, 9)_ = 18.10	**0.002**	0.668	*F*_(1, 9)_ = 0.97	0.351	0.097
	2nd half	1.78 ± 0.57^†^	1.93 ± 0.57^†^									
Breathing frequency (Breaths/min)	1st half	38 ± 8	39 ± 9	*F*_(1, 9)_ = 3.14	0.110	0.259	*F*_(1, 9)_ = 0.44	0.524	0.047	*F*_(1, 9)_ = 2.99	0.118	0.250
	2nd half	38 ± 9	40 ± 9									
Respiratory exchange ratio	1st half	0.99 ± 0.03	1.00 ± 0.03	*F*_(1, 9)_ = 1.28	0.287	0.125	*F*_(1, 9)_ = 148.49	**<** **0.001**	0.943	*F*_(1, 9)_ = 0.93	0.360	0.094
	2nd half	0.92 ± 0.04^†^	0.93 ± 0.05^†^									
Ventilatory equivalent for O_2_	1st half	30.0 ± 3.0	33.0 ± 3.4^*^	*F*_(1, 9)_ = 26.05	< 0.001	0.743	*F*_(1, 9)_ = 19.18	**0.002**	0.681	*F*_(1, 9)_ = 0.09	0.765	0.010
	2nd half	28.0 ± 2.9^†^	31.3 ± 3.2^†^									
Ventilatory equivalent for CO_2_	1st half	30.6 ± 3.0	33.2 ± 3.6^*^	*F*_(1, 9)_ = 22.21	**0.001**	0.712	*F*_(1, 9)_ = 0.01	0.945	0.001	*F*_(1, 9)_ = 0.51	0.494	0.053
	2nd half	30.4 ± 3.3	33.5 ± 3.0^*^									
End-tidal O_2_ partial pressure (mmHg)	1st half	90.2 ± 2.1	92.0 ± 2.3^*^	*F*_(1, 9)_ = 11.58	**0.008**	0.563	*F*_(1, 9)_ = 11.30	**0.008**	0.557	*F*_(1, 9)_ = 2.08	0.183	0.188
	2nd half	87.8 ± 2.9^†^	90.6 ± 3.3^*^									
End-tidal CO_2_ partial pressure (mmHg)	1st half	29.3 ± 2.7	27.2 ± 2.4^*^	*F*_(1, 9)_ = 19.56	**0.002**	0.685	*F*_(1, 9)_ = 5.63	**0.042**	0.385	*F*_(1, 9)_ = 0.93	0.360	0.094
	2nd half	30.3 ± 3.3^†^	27.6 ± 2.3^*^									
Heart rate (Beats/min)	1st half	153 ± 9	156 ± 9	*F*_(1, 9)_ = 1.91	0.200	0.175	*F*_(1, 9)_ = 0.04	0.855	0.004	*F*_(1, 9)_ = 0.35	0.569	0.037
	2nd half	153 ± 8	156 ± 9									
SpO_2_ (%)	1st half	95 ± 1	95 ± 1	*F*_(1, 9)_ = 3.08	0.113	0.255	*F*_(1, 9)_ = 2.06	0.185	0.186	*F*_(1, 9)_ = 0.00	0.972	0.000
	2nd half	94 ± 1	95 ± 1									

Bold values indicate statistical significance. Data are means ± SD. O_2_, oxygen; CO_2_, carbon dioxide; SpO_2_, peripheral oxygen saturation; ANOVA, analysis of variance; F, F-statistic from repeated-measures ANOVA; ηp^2^, partial eta squared; SD, standard deviation.

* *p* < 0.05 vs. placebo;^†^*p* < 0.05 vs. first half.

### Blinding efficacy and adverse-event observations

Blinding efficacy was assessed by asking participants to identify the treatment after each trial. Participants correctly guessed the assigned intervention in 11 out of 20 total trials (55%). Chi-square analysis revealed that the proportion of correct guesses was not significantly different from chance level (50%; χ^2^ = 0.10, *p* = 0.752), supporting adequate blinding of the chewing-gum interventions.

## Discussion

To our knowledge, this is the first study to demonstrate that caffeinated chewing gum enhances prolonged repeated-sprint performance under hypoxic conditions. The main findings were that, compared with placebo, caffeinated gum increased total work done, peak power output, and mean power output during both halves of the IST. These performance improvements were accompanied by enhanced cerebral oxygenation and greater muscle reoxygenation capacity. These findings support our hypothesis that caffeinated chewing gum would enhance prolonged repeated-sprint performance in hypoxia, accompanied by enhanced cerebral oxygenation and muscle reoxygenation capacity.

Our results demonstrated that caffeinated chewing gum increased total work done compared with placebo during both halves of the IST, with improvements of 4.4 and 2.9% in the first and second halves, respectively [*g*_*z*_ = 1.14, 95% CI (0.34, 1.93); *g*_*z*_ = 0.94, 95% CI (0.20, 1.69); Figure 1]. This finding is consistent with our previous study using the intermittent high-intensity exercise protocol (two 40-min halves) and 6 mg/kg caffeine capsules under hypoxic conditions, which reported similar performance improvements (6.2%−5.3%; *g*_*z*_ = 0.71–0.64) (16). The enhanced performance is partially supported by higher peak and mean power outputs and elevated blood lactate concentrations (Figures 2 extbfand 3) [the underlying mechanisms have been described in detail in our previous study (16)]. Furthermore, perceptual responses did not differ between the caffeine and placebo conditions (Figure 4). Nevertheless, total work done was higher following caffeine ingestion, suggesting that performance improvements may occur independently of changes in perceived exertion and may instead reflect enhanced effort tolerance or delayed neuromuscular fatigue (22). These findings suggest that caffeinated chewing gum can effectively deliver caffeine-mediated ergogenic effects during IST in hypoxia, extending previous evidence obtained with capsule-based caffeine supplementation. It should be noted that, although these effects represent large effect magnitudes, the relatively wide 95% CIs indicate limited precision of the estimates due to the small sample size. Thus, the observed point estimates should be interpreted cautiously and not considered definitive representations of stable population-level. In addition, both the present gum-based and previous capsule-based studies demonstrated substantial improvements in repeated-sprint performance under hypoxic conditions. Future head-to-head trials evaluating both delivery forms within the same cohort are warranted to directly compare their ergogenic efficacy.

Previous studies conducted under hypoxic conditions have demonstrated that reduced oxygen availability limits muscle reoxygenation during recovery periods between sprints (ΔReoxy), contributing to the progressive decline in repeated-sprint performance (6). Our results demonstrated that caffeinated chewing gum increased muscle ΔReoxy compared to placebo during the IST. The enhanced muscle ΔReoxy response may be partly explained by caffeine-induced increases in minute ventilation during both sprint and recovery periods of the IST (Tables 2 extbfand 3). Enhanced ventilatory responses may improve pulmonary oxygen exchange, potentially increasing oxygen availability within skeletal muscle during repeated-sprint exercise (45, 46). Furthermore, the enhanced muscle ΔReoxy may also be associated with caffeine-induced improvements in peripheral vascular function, potentially mediated by increased nitric oxide availability and modulation of vascular smooth muscle tone, which may enhance local blood oxygen availability in active muscle (47–49). Given that PCr resynthesis during recovery is highly dependent on oxygen availability (50, 51), enhanced muscle ΔReoxy may be associated with greater phosphagen restoration and preserved repeated-sprint performance under hypoxic conditions. However, the greater external work and power output observed with caffeine (Figures 1 extbfand 2) may also have increased oxygen extraction demands in the active muscles, meaning that the higher muscle [HHb] and larger peak-to-nadir HHb amplitude (ΔReoxy) could reflect greater metabolic demand rather than improved O_2_ delivery *per se*. Future studies are required to further clarify this mechanistic uncertainty.

Previous studies have suggested that reductions in cerebral oxygenation may contribute to impaired repeated-sprint performance under hypoxic conditions, potentially affecting central regulation during repeated high-intensity efforts (9). In the present study, caffeinated chewing gum increased cerebral oxyhemoglobin ([O_2_Hb]) and total hemoglobin ([THb]) compared with placebo during both halves of the IST (Table 1). Notably, caffeine increased minute ventilation while concurrently reducing end-tidal CO_2_, a physiological response that has been associated with cerebral vasoconstriction and reduced cerebral blood flow (52). These findings may reflect the combined influence of caffeine-induced central activation and its potential vascular effects (53–55); however, the underlying mechanisms remain uncertain. It should be noted that cerebral oxygenation was assessed using a single prefrontal NIRS optode pair without a short-separation channel, which cannot fully exclude contamination from extracerebral blood flow (e.g., skin and scalp circulation). Furthermore, direct measurements of cerebral blood flow were not acquired in this investigation. Therefore, mechanistic interpretations of these observed changes should be approached with caution.

### Practical applications

Our findings indicate that caffeinated chewing gum (6 mg/kg) enhances prolonged repeated-sprint ability during an 80-min simulated team-sport protocol under moderate hypoxic conditions. Therefore, when time for nutritional preparation is limited during competition, athletes participating in intermittent high-intensity team sports, particularly those competing at moderate altitude, may consider caffeinated chewing gum as a caffeine delivery strategy to support repeated-sprint performance.

### Additional considerations

There are some limitations of this study that should be considered. First, the IST protocol with fixed recovery intervals may not fully replicate the variable recovery demands encountered during actual team-sport competition, which may limit the ecological validity of the present findings (56). Second, given that extreme altitudes (i.e., 5,000 m) may further activate regulatory pathways and induce premature muscle fatigue, potentially compromising caffeine's ergogenic effect (57), the combined effects of hypoxia and caffeine on IST across varying simulated altitudes remain poorly understood. Third, the NIRS devices were located on the vastus lateralis. Although this muscle is a key contributor to power production during cycling (58), it is still possible that the effect of caffeine on muscle oxygen saturation would be different in other leg muscles. Fourth, our sample size was relatively small (*n* = 10) and included both male and female athletes, which may limit the generalizability of our findings and preclude robust sex-specific subgroup analyses. Future studies with larger and well-balanced samples are warranted to elucidate potential sex-specific differences in physiological and performance responses to caffeine during hypoxic intermittent sprint exercise. Fifth, the absence of plasma caffeine measurements precludes verification of the actual caffeine dose released from the gum and subsequently absorbed systemically. Sixth, the lack of a capsule comparator prevents us from determining whether the observed effects are specific to the gum formulation or attributable to caffeine itself. Seventh, NIRS measurements may be susceptible to extracerebral contamination from superficial tissues (e.g., skin and scalp blood flow), which could influence estimates of cerebral oxygenation despite the precautions taken to minimize this interference. Finally, given that multiple statistical comparisons were performed across performance, oxygenation, and gas exchange variables, the possibility of type I error cannot be excluded.

## Conclusion

Caffeinated chewing gum (6 mg/kg) enhances prolonged repeated-sprint ability under normobaric hypoxia, with enhancements in cerebral oxygenation and muscle reoxygenation capacity. These findings highlight caffeinated chewing gum as a caffeine delivery strategy for supporting high-intensity intermittent performance in team-sport athletes under hypoxic conditions.

## Data Availability

The original contributions presented in the study are included in the article/supplementary material, further inquiries can be directed to the corresponding author.
